# In Vitro Antimicrobial Synergistic Activity and the Mechanism of the Combination of Naringenin and Amikacin Against Antibiotic-Resistant *Escherichia coli*

**DOI:** 10.3390/microorganisms12091871

**Published:** 2024-09-11

**Authors:** Lankun Yi, Mingze Cao, Xu Chen, Yubin Bai, Weiwei Wang, Xiaojuan Wei, Yuxiang Shi, Yongying Zhang, Tenghe Ma, Zhen Zhu, Jiyu Zhang

**Affiliations:** 1 College of Life Science and Food Engineering, Hebei University of Engineering, Hanshan District, Handan 056038, China; ylankun@163.com (L.Y.); caomignze@126.com (M.C.); 13231656156@163.com (X.C.); syx1122024@163.com (Y.S.); zyy1122024@163.com (Y.Z.); matenghe@126.com (T.M.); 2 Lanzhou Institute of Husbandry and Pharmaceutical Sciences, Lanzhou 730050, China; byb1122024@163.com (Y.B.); www1120704@163.com (W.W.); wxj1122024@163.com (X.W.); 3 Key Laboratory of Veterinary Pharmaceutical Development, Chinese Academy of Agricultural Sciences, Ministry of Agriculture, Lanzhou 730050, China; 4 Key Laboratory of New Animal Drug Project of Gansu Province, Lanzhou 730050, China

**Keywords:** *Escherichia coli*, synergistic effect, naringenin, amikacin, antibacterial mechanism

## Abstract

Bacterial drug resistance is becoming an increasingly serious problem, and the development of antibacterial synergists is urgently needed. Combining existing antibiotics with promising nonantibiotic agents is one strategy that has been shown to be effective at overcoming the widespread emergence of antibiotic-resistant pathogens. In this study, we investigated the antibacterial activities and mechanism of naringenin (NG) combined with amikacin (AMK) against multidrug-resistant *Escherichia coli* (*E. coli*). We first measured the fractional inhibitory concentration (FIC) of NG combined with antibiotics via the checkerboard method. The results indicated that the combination of NG and AMK had a synergistic effect on *E. coli* ATCC 25922 and *E. coli* C7F3. In addition, this synergistic effect was verified by time-kill assays. Moreover, scanning electron microscopy (SEM) was used to observe cell morphology. The results showed that the cell wall of *E. coli* was destroyed. Furthermore, we assessed the leakage of alkaline phosphatase (AKP), K^+^, and protein. The extracellular AKP activity increased after the combinational group of 1/2MIC NG and 1/2MIC AMK, suggesting an impairment in cell wall permeability. An increase in the leakage of intracellular K^+^ and protein indicated an increase in cell inner membrane permeability. These results revealed that NG and AMK inhibited *E. coli* by damaging cell walls and membranes. In addition, PI uptake rapidly increased after treatment with NG and AMK. Confocal laser scanning microscopy (CLSM) revealed that NG caused cell wall and cell membrane damage in *E. coli*. In summary, our results provide a new strategy for responding to the development of *E. coli* drug resistance.

## 1. Introduction

Antibiotic treatment is the main therapeutic strategy used to fight bacterial pathogens in humans and animals [1]. However, the excessive use of antibiotics introduces a selective pressure that is becoming responsible for resistance or even multidrug resistance characteristics in some bacterial populations [2]. The increase in antibiotic resistance in pathogenic bacteria jeopardizes the value of antibiotics for reducing the morbidity and mortality related to bacterial infection, which has become a global concern [3]. *Escherichia coli* (*E. coli*) is one of the reservoirs of antibiotic resistance genes in the gastrointestinal tract and shares resistance determinants among different microorganisms [4]. Moreover, the challenge of the development of novel antibiotics and increasing antimicrobial resistance have brought unprecedented difficulties to clinical therapeutic options [5,6]. Thus, the identification and development of novel antibacterial compounds and new strategies that can combat resistance to well-established therapeutic agents are urgently needed.

Treatment for *E. coli* infection has become increasingly complicated because of the emergence of resistance to most first-line antimicrobial agents (such as trimethoprim-sulfamethoxazole and ciprofloxacin) [7]. One solution is the search for synergistic combination therapies between natural compounds and antibiotics, which would enhance antimicrobial therapy and enable the lowering of therapeutic doses. Throughout human history, plants have traditionally been used for various medicinal purposes and are primarily used to treat various microbial infections [8]. Flavonoids, which are enriched in several secondary metabolites in most plants, have been found to be effective antimicrobial substances against a wide variety of microorganisms and have shown potential for use in combination with antibiotics against resistant bacteria [9,10]. Previous studies have shown that flavonoids can produce synergistic antibacterial activity with different antibiotics by destroying the bacterial cell wall, changing membrane permeability, inhibiting biofilm formation, and other functional mechanisms [11]. In the study by Qu et al., when they treated cells with quercetin, in the presence of tetracycline, the levels of β-galactosidase and alkaline phosphatase were significantly elevated, indicative of cellular membrane disruption. This disruption was further illustrated using a PI uptake assay. This study showed that combination therapy produces profound ultrastructural changes, leading to an increase in permeability and a weakening of the cell envelope. Zhou et al. confirmed in their research that most experimental strains’ ability to build biofilms could be successfully inhibited by the combination of colistin and kaempferol [12,13].

Flavonoids are secondary metabolites that are very abundant in plants, fruits, and seeds. In humans, these compounds are associated with a large range of health benefits arising from their bioactive properties, such as anti-inflammatory, anticancer, anti-aging, cardio-protective, neuroprotective, immunomodulatory, antidiabetic, antibacterial, antiparasitic, and antiviral properties [14]. The antimicrobial activity of flavonoids against a wide variety of microorganisms has been demonstrated by in vitro tests [15]. Naringenin, a natural dihydroflavonoid widely found in food and used in traditional Chinese medicine, possesses extensive pharmacologic properties, including anti-inflammatory, free radical scavenging, and antioxidant activities, as demonstrated by numerous investigations [16,17,18]. However, previous studies investigated the potential antibiotic synergy between NG and antibiotics, but the specific mechanism is not clear.

This study aimed to investigate the effect of an antibacterial synergy between NG and AMK against multidrug-resistant *E. coli* and the potential underlying mechanism. Herein, we assessed in vitro antimicrobial synergist activities by using MICs, checkerboard tests, and time-kill assays. The mechanism was further studied by determining the permeability and integrity of the cell walls and membranes. 

## 2. Materials and Methods

### 2.1. Materials and Bacterial Strains

*E. coli* ATCC 25922 was purchased from the American Type Culture Collection. *E. coli* strains C7F3, C3F1, C5F3, B3E2, B5E1, A3E2, and A3F1 were isolated and preserved in our laboratory. Mueller Hinton Broth (MHB, Guangdong Huankai Microbial Sci.&Tech. Co., Ltd., Guangzhou, China) and Mueller Hinton Agar (MHA) media (Guangdong Huankai Microbial Sci.&Tech. Co., Ltd., Guangzhou, China) were used to cultivate all *E. coli* strains. NG was purchased from Shanghai Aladdin Biochemical Technology Co., Ltd. (Shanghai, China). Tetracycline, ciprofloxacin, meropenem, tigecycline, polymyxin, ceftriaxone, amikacin, and kanamycin were purchased from Beijing Solarbio Science & Technology Co., Ltd. (Beijing, China). NG was dissolved in dimethyl sulfoxide (DMSO). Tetracycline, ciprofloxacin, meropenem, tigecycline, polymyxin, ceftriaxone, amikacin, and kanamycin were dissolved in distilled water.

### 2.2. Antimicrobial Activity

#### 2.2.1. Determination of MIC and MBC

The broth microdilution method was performed according to the CLSI guidelines to determine the MICs of NG and eight common antibiotics against the tested *E. coli* strains [19]. Briefly, serial twofold dilutions of NG and different antibiotics were prepared and added to sterile 96-well plates, and DMSO was used as a control. Finally, 100 μL of bacterial suspension (0.5 McFarland suspension diluted 100× to a density of 10^6^ CFU/mL) was added. After culturing at 37 °C for 18–24 h, the MIC was identified as the well with no bacterial growth by measuring the OD600.

The MBC was determined after the MIC reading, removing aliquots from wells where there was no visible growth (supra-inhibitory concentrations) and inoculating them on MHA plates. MBC is defined as the lowest concentration capable of causing complete inhibition of bacterial growth after 24 h at 37 °C [20].

#### 2.2.2. Antibiotic Synergism Tests

The synergistic interaction between NG and antibiotics against *E. coli* was investigated using the checkerboard method [21]. Briefly, 50 μL of NG and antibiotics were serially diluted 2-fold (range from 2 × MIC to 1/32 × MIC) in a horizontal and a vertical arrangement in 96-well microplates, respectively. Then, 100 μL of *E. coli* suspension (0.5 McFarland suspension diluted 100× to a density of 10^6^ CFU/mL) was added to the 96-well microplates to mix with these NG/antibiotics combinations and incubated at 37 °C for 24 h. The FICI was calculated to quantify the synergy between NG and antibiotics using the following formula:

FICI = (MIC of NG in combination)/(MIC of NG alone) + (MIC of antibiotics in combination)/(MIC of antibiotics alone)(1)

The FICI indices were interpreted as follows: synergy (FICI ≤ 0.5), partial synergy (0.50 < FICI < 1), additive (=1), indifferent (1 < FICI ≤ 4), and antagonism (>4.00) (Fatemi et al., 2020). All tests were performed in triplicate, and the error bar represents standard error of the mean.

#### 2.2.3. Time-Kill Curve Assays

To confirm the antibacterial effects of the combination of NG, the plate count method was applied and the growth inhibition of the tested bacterial strains was investigated [22]. The bacteria were grown to the logarithmic growth phase and diluted to 1 × 10^6^ CFU/mL. *E. coli* cells (1 × 10^6^ CFU/mL) were incubated under four different treatment conditions: a group with no treatment served as a control, 1/2MIC NG, 1/2MIC AMK, and 1/2MIC NG+1/2MIC AMK. During incubation, 50 µL aliquots were removed from each tube at several time intervals of 0, 4, 8, 12, and 24 h and diluted serially using 450 µL of sterile saline solution (1:10). From each dilution, 20 µL was inoculated on MHA plates and incubated for 24 h at 37 °C. Then, the number of viable colonies was counted only from the plates containing between 30 and 300 colonies to evaluate the antibacterial effect of NG. The experiment was repeated in triplicate, and the error bar represents standard error of the mean.

### 2.3. The Antibacterial Mechanism

#### 2.3.1. SEM

The samples were processed according to published methods with slight modifications [23]. SEM was applied to observe the morphological changes in indicators after treatment with 1/2MIC NG, 1/2MIC AMK, or 1/2MIC NG+1/2MIC AMK at 37 °C for 6 h, and untreated *E. coli* served as a control. Bacterial cells after treatment of NG or AMK underwent centrifugation at 8000 rpm for 8 min and washed 2 times with sterile PBS. Washed bacterial pellets were fixed in 3.0% glutaraldehyde for 48 h and again washed with phosphate-buffered saline. Following washing, the bacterial pellets were dehydrated with graded ethanol (30%, 50%, 70%, 80%, 90%, 95%, and 100%), and each elution time was 15 min. Isoamyl acetate was used to replace ethanol as an intermediate medium in the samples. The processed samples were analyzed using SEM (JSM-6701F, Hitachi, Japan).

#### 2.3.2. Cell Wall Permeability

AKP is a periplasmic enzyme whose activity is undetectable in the extracellular environment when the bacterial cell wall is intact [24]. Therefore, the integrity of the cell wall can be reflected by the change in extracellular AKP content. The extracellular AKP assay experiments were carried out as previously reported with minor modifications [25]. *E. coli* ATCC 25922 and C7F3 were cultured in MHB at 37 °C for 12 h and washed three times with PBS. The cultures were adjusted to match the turbidity of the 0.5 McFarland turbidity standard; *E. coli* cells were treated with 1/2MIC NG, 1/2MIC AMK, or 1/2MIC NG+1/2MIC AMK at 37 °C for 6 h, and untreated *E. coli* served as the control. Then the testing objects were centrifuged at 3500 rpm for 10 min and the content of AKP in the supernatant was measured by the AKP kit assay (Nanjing Jiancheng Bioengineering Institute, Nanjing, China) according to the manufacturer’s instructions.

#### 2.3.3. Inner Membrane (IM) Permeability

##### Detection of K^+^ and Protein Leakage

Intracellular K^+^ leakage [26] and protein levels [27] indicate the permeability of the cell membrane. Therefore, the K^+^ and protein content in the supernatant of the bacterial suspension was determined. *E. coli* ATCC 25922 and C7F3 were cultured in MHB at 37 °C for 12 h and washed three times with PBS. The cultures were adjusted to match the turbidity of the 0.5 McFarland turbidity standard. Then, *E. coli* cells were treated for 6 h with 1/2MIC NG, 1/2MIC AMK, or 1/2MIC NG+1/2MIC AMK, and untreated *E. coli* served as the control. After the treatment was completed, the supernatant was centrifuged for the detection of protein and K^+^. The protein concentration of the supernatant was quantified using a BCA protein assay kit (Meilunbio, Dalian, China). The K^+^ concentration was measured using a potassium (K^+^) turbidimetric assay kit (Elabscience, Wuhan, China) according to the manufacturer’s protocol [28].

##### IM Permeability of *E. coli* Was Determined Using CLSM

To verify visually the result of K^+^ and protein leakage, inner membrane damage was further detected using confocal laser scanning microscopy (LSM800, Zeiss, Jena, Germany) as described by Sun et al. [29]. Cells treated with 1/2MIC NG, 1/2MIC AMK, or 1/2MIC NG + 1/2MIC AMK, and undrugged *E. coli* cells were collected and dyed with 100 μM 5,6-carboxyfluorescein diacetate (cFDA) and 15 μM propidium iodide (PI) in the dark. The stained cells were incubated in the dark at 37 °C for 15 min and then washed with PBS. The washed samples were dropped onto a glass slide, covered with a coverslip, and then observed using CLSM at a magnification of 1000×. The 5,6-cFDA was detected under a 488 nm laser light, and PI was detected at 565 nm.

### 2.4. Statistical Analysis

The data analysis was performed using GraphPad Prism 8.0 software and presented as the mean ± standard deviation (SD). For two-group comparisons, data were analyzed using Student’s *t*-test, and for multiple comparisons, *p* values were measured using one-way ANOVA. A statistically significant difference was considered as *p* < 0.05.

## 3. Results

### 3.1. Antibacterial Activity of NG

We explored the antibacterial activities of eight antibiotics against eight strains of *E. coli* using NG alone and in combination. According to the results of the microdilution test, the bacterial strains used in the study (except for ATCC 25922) were all resistant strains, of which C7F3 and A3E2 were multi-drug resistant (MDR) strains (Table 1). Multidrug-resistant bacteria refer to microorganisms that are simultaneously resistant to three types or three or more types of antibacterial drugs with different mechanisms [30]. As shown in Table 2, the MICs of NG against all seven strains were ≥1 mg/mL, and the MBCs were ≥2 mg/mL, indicating that NG alone did not exhibit good antibacterial activity against ATCC 25922 or the other seven clinically tested *E. coli* strains. Therefore, we sought to evaluate the synergistic effect of NG and antibiotics by determining the FIC. The first non-turbid well in each row and column was used to calculate the FIC index. The results showed that NG can act as a synergistic antibacterial agent with AMK for *E. coli* ATCC 25922, *E. coli* C7F3, *E. coli* C3F1, *E. coli* B3E2, and *E. coli* A3F1 (Table 3), with FIC values of 0.3125, 0.1875, 0.375, 0.5, and 0.375, respectively. Therefore, we selected NG and AMK against *E. coli* ATCC 25922 and *E. coli* C7F3 for the time-kill assay.

### 3.2. The Combination of NG and AMK Shows Potent Bactericidal Activity Against E. coli

**Figure 1:**
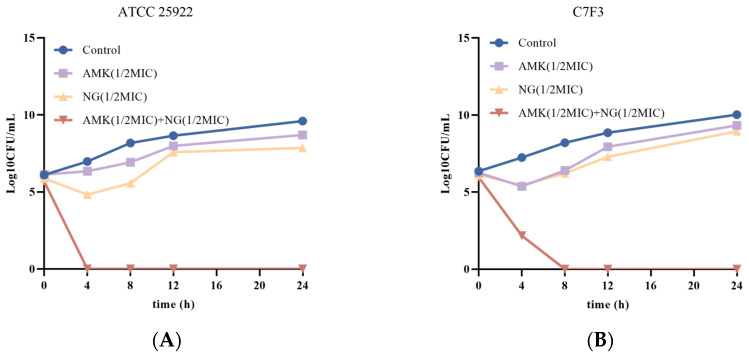
Time-kill curves of 1/2MIC NG and 1/2MIC AMK alone and in combination against *E. coli* ATCC 25922 (**A**) and *E. coli* C7F3 (**B**). Each value is presented as the mean ± SD (n = 3).

Based on the findings of the combined effect of NG and AMK on *E. coli*, we conducted a series of time-kill studies using *E. coli* strains C7F3 and ATCC 25922 at a concentration of 1/2MIC AMK and 1/2MIC NG. The time-kill curves for *E. coli* ATCC 25922 and *E. coli* C7F3 are shown in Figure 1A,B. The results showed that the monotherapy group inhibited the growth of *E. coli* ATCC 25922 but did not completely kill *E. coli*. Additionally, in the combinational group of 1/2MIC NG and 1/2MIC AMK, bacteria were killed over the course of 4 hours. Similarly, in the case of *E. coli* C7F3, although there was no significant difference in the bactericidal effect between NG and the control group, 1/2MIC NG in combination with 1/2MIC AMK resulted in faster killing, and the strains treated with the combination exhibited a significant reduction in viable cells. In summary, 1/2MIC NG and 1/2MIC AMK can effectively kill *E. coli* ATCC 25922 and *E. coli* C7F3 when used together.

### 3.3. The Combination of NG and AMK Causes Changes in the Morphology of E. coli

**Figure 2:**
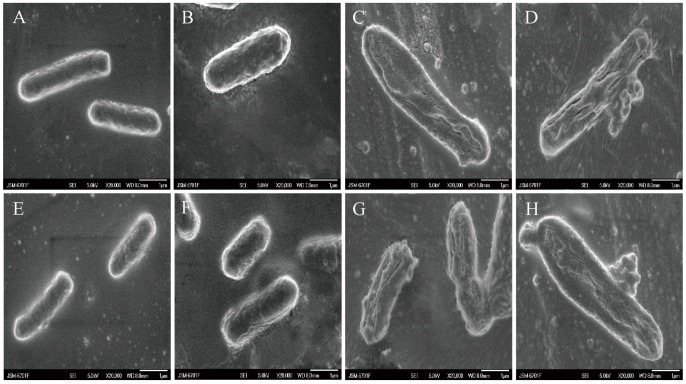
Effects of 1/2MIC NG and 1/2MIC AMK alone or in combination on the morphology of *E. coli* ATCC 25922 (**A**–**D**) and *E. coli* C7F3 (**E**–**H**) cells. (**A**,**E**): control; (**B**,**F**): 1/2MIC AMK; (**C**,**G**): 1/2MIC NG; and (**D**,**H**): 1/2MIC NG + 1/2MIC AMK. Magnification = 20,000×. Ten visual fields were observed for each replicate and representative images were selected.

Figure 2 shows SEM micrographs of untreated *E. coli* ATCC 25922 and C7F3 (Figure 2A,E), *E. coli* ATCC 25922 and C7F3 treated with 1/2MIC AMK (Figure 2B,F), *E. coli* ATCC 25922 and C7F3 treated with 1/2MIC NG (Figure 2C,G), *E. coli* ATCC 25922 and C7F3 treated with 1/2MIC NG, and 1/2MIC AMK (Figure 2D,H). The bacterial cells in the control group exhibited a complete morphology, clear outlines, were clean and plump, and had a smooth surface (Figure 2A,E). Similarly, the morphology of the bacteria in the AMK-treated group was similar to that of the bacteria in the control group (Figure 2B,F). The NG-treated bacterial cells, which were shriveled, wrinkled, distorted, and deformed, are shown in Figure 2C,G, where bacteria stretched and became longer, and the surface collapsed. Moreover, compared with that in the NG-treated group, the combined drug groups exhibited greater damage to the cell morphology. As shown in Figure 2D,H, among *E. coli* ATCC 25922 and *E. coli* C7F3, the combined bacterial samples showed surface alterations, with membrane disruption and apparent leakage.

### 3.4. The Combination of NG and AMK Damages the Cell Wall

**Figure 3:**
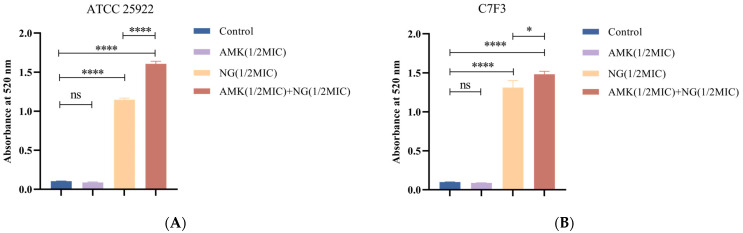
Effects of 1/2MIC NG and 1/2MIC AMK alone or in combination on AKP leakage of *E. coli* ATCC 25922 (**A**) and *E. coli* C7F3 (**B**). Each value is presented as the mean ± SD (*n* = 3). ^ns^ *p*-value > 0.05, * *p*-value < 0.05, and **** *p*-value < 0.0001.

AKP is a periplasmic enzyme. A large amount of intracellular AKP leakage can indicate that the integrity of the cell wall has been destroyed. Therefore, the integrity of the cell wall can be reflected by the change in AKP content in the extracellular environment. As shown in Figure 3A, the amount of extracellular AKP released in the NG-treated group was greater than that in the control group (*p* < 0.0001), and that in the combined treatment group was significantly greater than that in the NG-treated group (*p* < 0.0001). As shown in Figure 3B, compared with the control group, the content of AKP in the NG-treated group was significantly increased (*p* < 0.0001), and the content of AKP in the combined treatment group was significantly Increased compared with the NG-treated group (*p* < 0.05). These results suggest that NG can destroy the cell wall of *E. coli* and lead to increased AKP leakage, and that NG can synergize with AMK to enhance the destruction of the cell wall.

### 3.5. The Combination of NG and AMK Damages the Inner Membrane (IM)

#### 3.5.1. Detection of Protein Leakage

**Figure 4:**
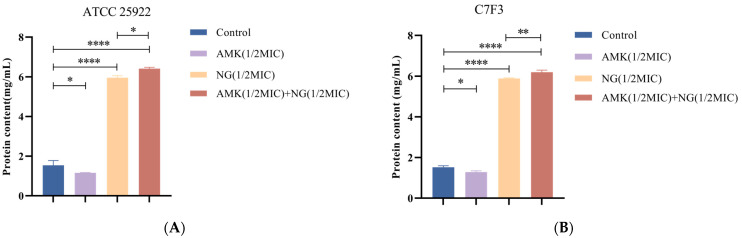
Effects of 1/2MIC NG and 1/2MIC AMK alone or in combination on protein leakage of *E. coli* ATCC 25922 (**A**) and *E. coli* C7F3 (**B**). Each value is presented as the mean ± SD (*n* = 3). * *p*-value < 0.05, ** *p*-value < 0.01, and **** *p*-value < 0.0001.

The permeability of the inner membrane was determined by measuring protein leakage. As shown in Figure 4, compared with those of the control, after the addition of 1/2MIC NG and 1/2MIC NG+1/2MIC AMK for 6 h, the protein release of *E. coli* ATCC 25922 increased by approximately 2.45-fold and 3.27-fold, respectively (Figure 4A), and the protein release of *E. coli* C7F3 increased by approximately 2.88-fold and 3.47-fold, respectively (Figure 4B). In addition, protein release decreased by approximately 0.28-fold and 0.25-fold with the addition of 1/2MIC AMK in *E. coli* ATCC 25922 and *E. coli* C7F3, respectively. This is because the mechanism of action of amikacin is to inhibit protein synthesis by ribosomes, which is consistent with the results of this experiment.

#### 3.5.2. Detection of K^+^ Leakage

**Figure 5:**
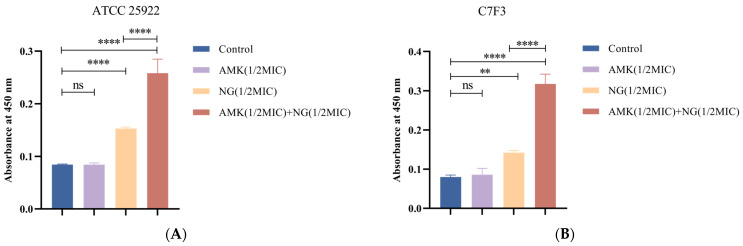
Effects of 1/2MIC NG and 1/2MIC AMK alone or in combination on K^+^ leakage of *E. coli* ATCC 25922 (**A**) and *E. coli* C7F3 (**B**). Each value is presented as the mean ± SD (n = 3). ^ns^ *p*-value > 0.05, ** *p*-value < 0.01, and **** *p*-value < 0.0001.

The K^+^ leakage of the cellular membrane is closely related to its integrity and stability. Therefore, membrane permeability was analyzed by measuring the leakage of potassium ions. As shown in Figure 5A,B, after treatment with 1/2MIC NG and 1/2MIC NG+1/2MIC AMK, the potassium ion concentration outside of the *E. coli* cell increased significantly (*p* < 0.05). K^+^ leakage increased by approximately 0.82-fold and 0.71-fold after NG-treatment, respectively, and increased by approximately 2.06-fold and 2.81-fold after NG+AMK, respectively. K^+^ leakage after the combined treatment was greater than that after the NG treatment. In summary, we can conclude that combined treatment with NG and AMK can effectively damage the cell inner membrane of *E. coli* and cause a large amount of K^+^ to leak.

#### 3.5.3. CLSM Examinations

**Figure 6:**
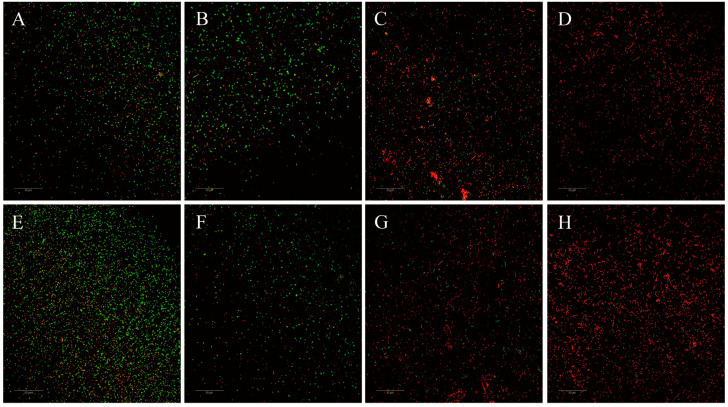
CLSM images of *E. coli* ATCC 25922 and *E. coli* C7F3 treated with 1/2MIC NG and 1/2MIC AMK alone or in combination. (**A**,**E**): control; (**B**,**F**): 1/2MIC AMK; (**C**,**G**): 1/2MIC NG; and (**D**,**H**): 1/2MIC NG + 1/2MIC AMK. Scale Bar: 50 µm.

The IM permeability of *E. coli* cells was examined using CLSM with 5,6-cFDA and PI. 5,6-cFDA cannot fluoresce; however, it can readily diffuse across intact cell membranes and be converted into carboxyfluorescein. PI cannot enter intact cell membranes but can cross compromised cell membranes and emit red fluorescence. Thus, intact cells emit green fluorescence, while damaged cells emit red fluorescence. Additionally, the fluorescence of PI and 5,6-cFDA overlaps and appeared yellow when the membrane was damaged but esterase activity also existed. The confocal laser scanning microscopy results are presented in Figure 6 and Figure S1. The red/green ratios of the NG-treated groups (Figure 6A,E) increased compared with those of the untreated group (Figure 6D,H), and the red/green ratios in the combined treatment groups (Figure 6B,F) were much greater than those in the NG-treated group. In contrast, there was no significant difference between the AMK-treated group (Figure 6C,G) and the control group. This finding indicates that treatment with NG caused membrane damage, and the combination treatment further aggravated the damage.

## 4. Discussion

Among the resistant bacteria, *E. coli* is the most common gram-negative bacterial pathogen, presenting both clinical and epidemiological challenges [31]. In the last decade, the overuse of antibacterial drugs has led to an increase in multidrug-resistant strains [32], and antibiotics have lost their effectiveness over time. Some empirical studies have shown that traditional Chinese medicine can offer several advantages, such as less toxic bioactive compounds, biodegradability, and a widespread spectrum of secondary metabolites, which make it a common alternative source of antibiotics [33]. The concomitant use of antibiotics and natural products is a pragmatic way to overcome antimicrobial drug resistance, and elucidation of their synergistic mechanism is the key to direct clinical use and new agent development [34]. However, no one has studied the antibacterial mechanism of naringenin combined with antibiotics against *E. coli*. We evaluated the in vitro activity of NG in combination with other antibiotics and further explored the synergistic mechanism. In this study, we found that NG could improve sensitivity to AMK, and that NG had a synergistic effect with AMK against *E. coli*. Additionally, our results suggested that NG and AMK exerted bacterial killing effects on *E. coli* mainly by destroying the structure of the bacterial cell membrane and cell wall.

In this study, the antibacterial effect of NG alone and in combination with antibiotics against drug-resistant *E. coli* was tested in vitro, and the combination of NG and AMK effectively reduces the dosage of both and has a good bactericidal effect. This result was further confirmed using a time-killing assay.

The possible mechanism of NG was identified using SEM and membrane permeability and integrity tests. We used SEM to observe the prominent loss of cell shape and integrity. Thus, combined with previous studies, we speculate that NG and AMK may play a role in inhibiting and killing *E. coli* by damaging cell walls and cell membranes, thereby leaking cell contents.

The cell wall and membrane of gram-negative bacteria are important for maintaining the shape and structural integrity of bacterial cells. In some experiments, lactate AKP, K^+^, and protein release were used as indicators of cell wall and cell membrane damage [35,36]. Next, we examined changes in the above indicators. When the cell wall is damaged, AKP leaks out, leading to an increase in extracellular AKP activity. The experimental results showed that the AKP content in the combined treatment groups was significantly greater than that in the other groups, consistent with findings from other studies.

**Figure 7:**
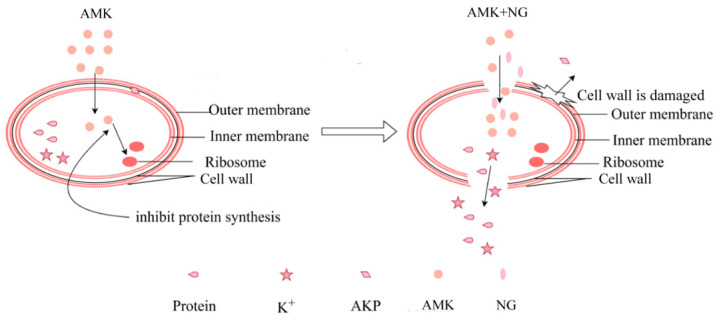
A model of NG and AMK combination therapy on *E. coli* cells. When AMK is used alone, the cell wall and cell membrane of drug-resistant bacteria are intact, making it difficult for AMK to enter the bacteria to exert its antibacterial effect. However, when NG is used in combination with AMK, NG will destroy the integrity of the *E. coli* cell wall and cell membrane, allowing AMK to smoothly enter the interior of the bacteria in large quantities, inhibit ribosomes from synthesizing proteins, and quickly kill drug-resistant *E. coli*.

In addition, K^+^ leakage and protein leakage of bacterial suspensions increased significantly after combination treatment [37]. When the cell membrane is damaged, its permeability increases. As a result, the cellular contents (proteins and K^+^) are released into the environment [38]. This further confirmed that the inner membrane of *E. coli* was damaged after the action of NG and AMK. We also found that the protein content of the AMK groups was lower than that of the control group. Combined with previous research, we think that this phenomenon is related to the antibacterial mechanism of AMK. AMK is a semisynthetic aminoglycoside that exerts its bacterial killing effects by interfering with intracellular protein synthesis [39]. AMK treatment inhibits *E. coli* protein synthesis, resulting in reduced protein leakage. CLSM was performed to determine the extent of cell membrane damage as measured by the uptake of PI. Based on these findings, we found that NG destroys the cell wall and membrane structure of *E. coli*, facilitating the entry of AMK into the cell and subsequent antibacterial effects. However, further research is needed to analyze its potential clinical application. We will apply this combination to chicken in the future to further determine its antibacterial effect in vivo. A detailed schematic diagram of the antibacterial mechanism is shown in Figure 7.

## 5. Conclusions

We found that NG can greatly enhance the sensitivity of *E. coli* to AMK. Moreover, research has indicated that NG destroys the integrity of the cell membrane and cell wall and leads to a substantial amount of AMK entering bacterial cells to exert bacteriostatic effects. More importantly, NG combined with AMK can reduce the dose of AMK needed, thereby reducing its side effects (ototoxicity and nephrotoxicity) and maintaining the maximum therapeutic effect. Our findings provide a potential treatment strategy involving the use of existing antibacterial agents in combination with adjuvants to suppress infections caused by *E. coli*. Combining NG with AMK can reduce the dosage of antibiotics and plant extracts, enhance the antibacterial activity of antibiotics, and restore the sensitivity of drug-resistant bacteria to antibiotics. This is an effective strategy to enhance the effect of antibiotics. Although the production cost of NG is low and the extraction method is environmentally friendly and highly efficient, more potential issues need to be considered for clinical application (such as the administration method, dosage, etc.). In the future, we will further study its cytotoxicity, bioavailability, and the stability of the pharmaceutical dosage form to determine its therapeutic effect on colibacillosis. Synergists are used in clinical treatment in combination with antibiotics to achieve the strategic goal of reducing and replacing antibiotics.

## Figures and Tables

**Table 1:** MICs of eight antibiotics against eight strains of *E. coli* (μg/mL).

	ATCC 25922	C7F3 *	C3F1	C5F3	B3E2	B5E1	A3E2 *	A3F1
Tetracycline	0.5 (S)	1024 (R)	512 (R)	512 (R)	512 (R)	1024 (R)	512 (R)	256 (R)
Ciprofloxacin	0.0039 (S)	4 (R)	0.125 (S)	0.5 (I)	0.25 (S)	0.03125 (S)	8 (R)	0.0625 (S)
Meropenem	0.03125 (S)	0.0078 (S)	0.015625 (S)	0.0625 (S)	0.03125 (S)	0.015625 (S)	16 (R)	0.0078 (S)
Tigecycline	0.03125 (S)	16 (R)	8 (R)	16 (R)	16 (R)	2 (S)	2 (S)	4 (I)
Polymyxin	0.5 (I)	0.5 (I)	0.25 (I)	1 (I)	0.25 (I)	0.25 (I)	1 (I)	0.125 (I)
Ceftriaxone	0.125 (S)	512 (R)	512 (R)	256 (R)	512 (R)	0.125 (S)	256 (R)	128 (R)
Amikacin	2 (S)	64 (R)	4 (S)	2 (S)	2 (S)	4 (S)	32 (I)	1 (S)
Kanamycin	1 (S)	256 (R)	8 (S)	4 (S)	4 (S)	1024 (R)	256 (R)	2 (S)

S, sensitive; I, intermediary; R, resistant; * MDR *E. coli.*

**Table 2:** MICs and MBCs of NG against eight strains of *E. coli* (mg/mL).

	MIC	MBC
ATCC 25922	1	2
C7F3	2	4
C3F1	2	8
C5F3	8	16
B3E2	2	4
B5E1	8	16
A3E2	2	4
A3F1	4	8

**Table 3:** FICs of NG combined with different antibiotics against *E. coli*.

	ATCC 25922	C7F3	C3F1	C5F3	B3E2	B5E1	A3E2	A3F1
Tetracycline	0.5625	1	1	0.5	0.625	1	1	1
Ciprofloxacin	1	1	0.75	1	1	0.625	0.53125	1
Meropenem	1	1	0.5	1	1	1	1	1
Tigecycline	1	0.5625	0.625	1	1	0.75	0.75	1
Polymyxin	0.5	1	1	1	0.75	0.5625	0.75	1
Ceftriaxone	1	0.75	1	0.5	1	1	1	0.625
Amikacin	0.3125	0.1875	0.375	0.75	0.5	0.75	0.625	0.375
Kanamycin	0.75	1	0.75	1	1	1	1	1

## Data Availability

The original contributions presented in the study are included in the article/Supplementary Material, further inquiries can be directed to the corresponding authors.

## Supplementary Materials

The following supporting information can be downloaded at: https://www.mdpi.com/article/10.3390/microorganisms12091871/s1. Figure S1: CLSM supplementary images.
