# Supplementary figures and images for: In Vitro Antimicrobial Synergistic Activity and the Mechanism of the Combination of Naringenin and Amikacin Against Antibiotic-Resistant Escherichia coli
Source: Microorganisms. 2024 Sep 11;12(9):1871. doi: 10.3390/microorganisms12091871 (PMC11433787; doi:10.3390/microorganisms12091871)

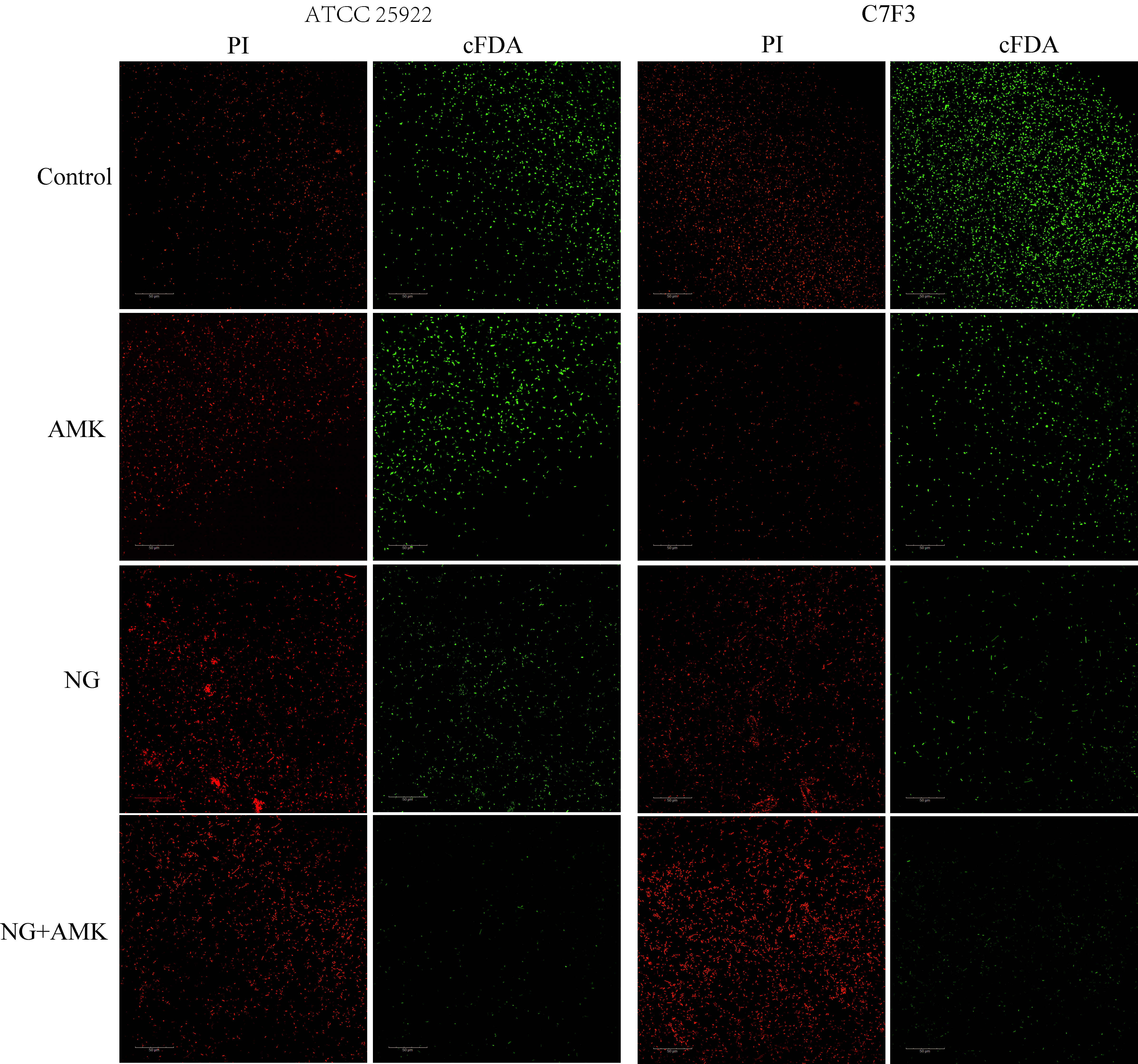

Supplement: Supplementary file 1 [file microorganisms-12-01871-s001.zip › microorganisms-3131446-supplementary Figure S1.tif]
